# Was Cajal right about sleep?

**DOI:** 10.1186/s12915-015-0178-5

**Published:** 2015-08-25

**Authors:** Matthew C F Tso, Erik D. Herzog

**Affiliations:** Department of Biology, Washington University in St. Louis, St. Louis, MO 63130 USA

## Abstract

Cajal’s careful observations of the anatomy of the nervous system led him to some lesser-known predictions about the function of glia as mediators of sleep. Reporting over 120 years later in *BMC Biology*, Bellesi et al. examine changes in gene expression and morphology of astrocytes with sleep. Their results provide support for and revisions to Cajal’s predictions.

See research article: doi: 10.1186/s12915-015-0176-7.

## Main text

**Fig. 1.**
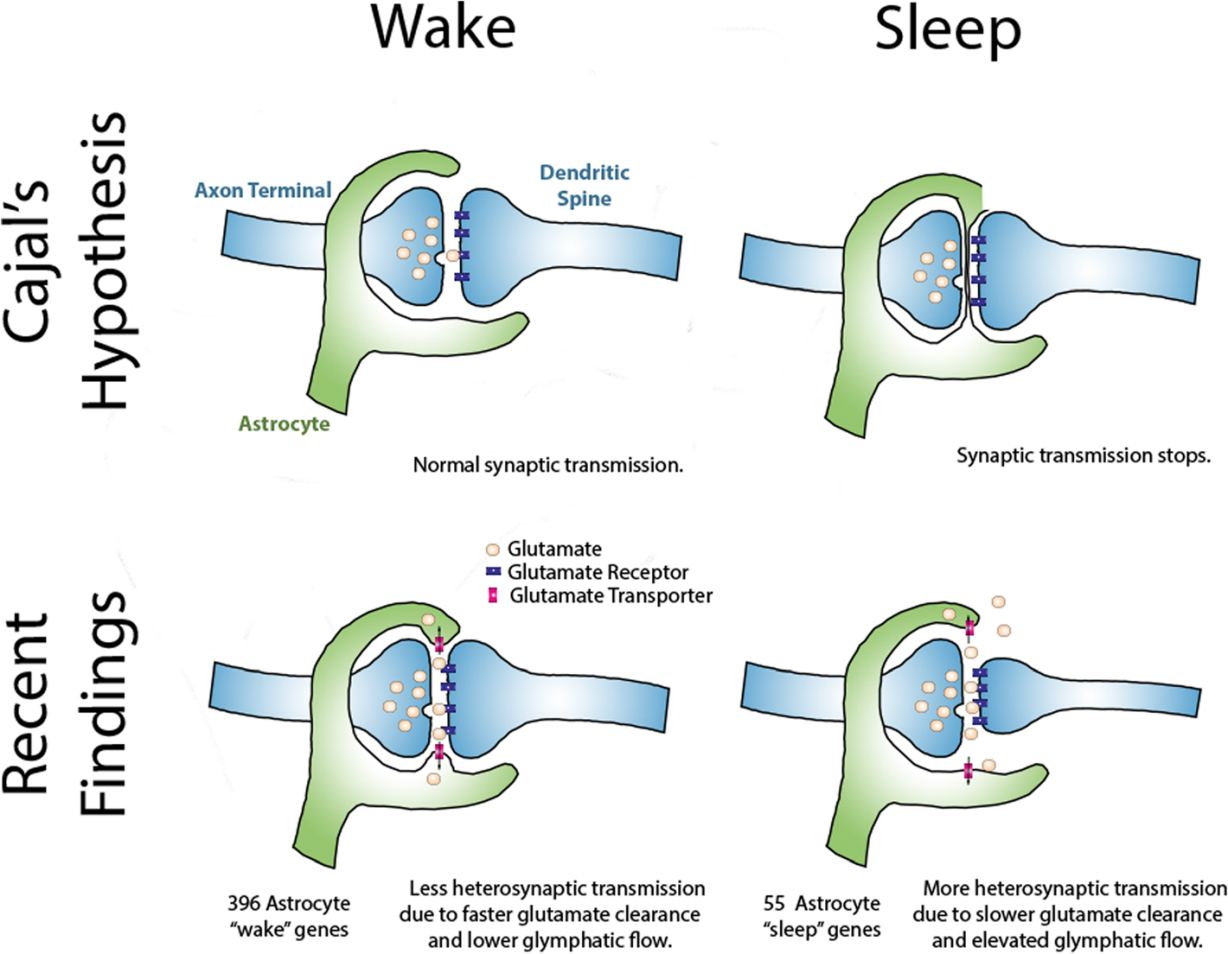
Historical and current understanding of the role astrocytes in sleep. *Top*: Cajal postulated that astrocytes retract their processes during wakefulness to allow normal synaptic transmission. During sleep, he reasoned, astrocyte processes invade the synapse to block synaptic transmission. *Bottom*: Recent findings revise this model to include astrocyte processes covering the synapse, presumably to enhance glutamate clearance during wake and retracting during sleep. This correlates with changes in gene expression in astrocytes (with ~400 transcripts up-regulated during wake and ~50 during sleep) and could relate to more glutamate spillover and increased glymphatic flow during sleep. Previous findings also showed dendritic spines grow with time awake (and greater sleep debt; reviewed in [3]). The model thus posits that changes in glial gene expression, morphology, and physiology may modulate synaptic transmission to promote sleep

The great neuroscientist Santiago Ramón y Cajal (1852–1934) studied neurons extensively and, also, glial cells — astrocytes in particular. Based on his observation that the length of astrocytic processes varies greatly between cells, he imagined that astrocytes dynamically extend and retract their processes. He boldly hypothesized, in 1895, that: (1) during sleep, endfeet of astrocytes invade the synaptic cleft to serve as a ‘circuit breaker’, pausing synaptic transmission and (2) during wakefulness, those endfeet retract, restoring synaptic transmission [1, 2] (Fig. 1).

The health benefits and evolutionary conservation of sleep have inspired many subsequent studies on the mechanisms that initiate, maintain, and modulate sleep (reviewed in [3]). Sleep deprivation impairs cognitive performance and, when prolonged for days to weeks, can cause seizures and death. How does the brain measure the need to sleep? What does sleep do for the brain?

In their article in *BMC Biology*, Bellesi et al. [4] investigate how gene expression and ultrastructural synaptic morphology vary with different sleep–wake states in the mouse brain. Using bacterial artificial chromosome-translating ribosome affinity purification (BAC-TRAP) followed by microarray analysis, they compare levels of translating mRNAs in cortical astrocytes (specifically, Aldh1L1-positive cells in cortex and striatum) and find 1.4 % of astrocyte transcripts increase or decrease with sleep compared with transcript levels during wakefulness and sleep deprivation. Surprisingly, in astrocytes, the number of genes uniquely expressed during waking (396 ‘wake genes’) drastically outnumbered the 55 sleep-associated genes. This differs from a similar study on oligodendrocytes, where the same group found similar numbers of both sleep and wake genes [5]. In astrocytes, they report genes involved in cell development and proliferation were enriched during sleep while genes related to metabolism and anatomical structure development were enriched during wakefulness. Among the genes with the greatest increase in transcript levels during wakefulness are a set that could mediate elongation of astrocytic processes, including *Trio*, *Synj2*, and *Gem*.

These gene hits led the authors to study sleep state-dependent changes in synaptic structure by serial block face scanning electron microscopy (SBF-SEM). In this method, researchers use SEM to image the surface of a tissue after each section is cut at less than 30 nm thick. This state-of-the-art technique generates nanometer-resolution, three-dimensional images of brain tissue and is particularly useful in investigating synaptic structure and tracing neuronal connections. They find that sleep deprivation (acute or chronic) increases the number of dendritic spines covered by astrocytic processes in layer II of the prefrontal cortex. Chronic sleep deprivation also extends the astrocyte coverage of neuronal spines, an intriguing observation that may relate to the reported increase in size and number of synapses during wakefulness in *Drosophila* and mice [3]. Thus, Cajal correctly predicted that astrocyte coverage of dendritic spines is state-dependent, although Bellesi et al. report astrocytic endfeet are closer to, rather than farther from, synapses during wake.

The movement of astrocyte processes may reflect a component of a sleep homeostat, measuring the accumulation of sleep debt during wake [1]. Astrocytes are well positioned to detect and signal sleep need — ubiquitous throughout the brain, able to integrate firing and metabolic activity of neurons, and able to modulate neural activity. Astrocyte physiology has been correlated repeatedly with sleep/wake state. To date, however, evidence to support the hypothesis that, with accumulating sleep debt, astrocytes feedback to neurons to increase sleep propensity, duration, and intensity remains correlative. We lack, for example, conclusive evidence that manipulations of cortical astrocytes result in sleep dysfunction [6, 7].

Why would synaptic coverage by astrocytes increase during wakefulness and further increase with accumulating sleep debt? Increased astrocytic coverage during wake could facilitate clearance of glutamate at synapses. Extracellular glutamate content in cortex and overall cortical firing rate are higher during wakefulness, requiring enhanced clearance of synaptic glutamate [8]. Since the synaptic cleft lacks glutamate-degrading enzymes, glutamate transporters in astrocyte endfeet must clear glutamate from the synapse fast enough to ensure temporal resolution of signal transduction. If this hypothesis is true, slower glutamate clearance could also provide feedback to neurons through extrasynaptic metabotropic glutamate receptors during sleep [8]. Retraction of astrocyte processes at night may lead to spillover of glutamate and facilitate heterosynaptic transmission and the synchronous firing characteristic of slow wave sleep. To bridge the gap between these recent ultrastructural observations and the proposed physiological mechanisms, future studies should assess glutamate uptake and clearance from extracellular space by astrocytes during wake and sleep. Care should be taken to consider differences that may exist between brain regions, neurotransmitters, and with the stages of sleep. For example, a recent report shows that loss of the GLT-1 glutamate transporter in astrocytes of the lateral habenula causes animals to spend more time in rapid eye movement (REM) sleep [9]. Finally, it will be exciting to learn if perturbation of the position of astrocyte endfeet modulates transmitter clearance and sleep regulation.

Apart from glutamate clearance, Bellesi et al.’s observations also provide support for the emerging hypothesis that sleep enhances performance of the glymphatic system. Increasing interstitial space by as much as 60 %, sleep appears to facilitate clearance of metabolites, including those that can lead to neurodegenerative diseases [10]. Astrocytes clearly play a role in the glymphatic system as mice lacking the astrocyte-specific water channel *Aqp4* show a twofold decrease in metabolite clearance [10]. However, it is unclear if or how *Aqp4* might modulate glial morphology, the interstitial or synaptic space and, ultimately, sleep. Therefore, future studies should seek the mechanistic links from decreased glial synaptic coverage to increased convective flow and sleep.

In 1911, Cajal wrote: “What is the function of glial cells in neural centers? The answer is still not known, and the problem is even more serious because it may remain unsolved for many years to come until physiologists find direct methods to attack it” [11]. More than a century later, molecular tools and physiological assays are becoming available to manipulate and monitor the role of astrocytes. Bellesi et al. take Cajal’s charge and place astrocytes as central to sleep.
